# ACT001 improved cardiovascular function in septic mice by inhibiting the production of proinflammatory cytokines and the expression of JAK-STAT signaling pathway

**DOI:** 10.3389/fphar.2023.1265177

**Published:** 2023-11-29

**Authors:** Zhen Peng, Xiaolong Lv, Xintong Wang, Ting Shang, Jing Chang, Khalid Salahdiin, Yue Guo, Zhisen Zhang, Ru Shen, Ming Lyu, Shuang He, Jian Yang, Yuefei Wang, Xiumei Gao, Yan Zhu, Yuxin Feng

**Affiliations:** ^1^ State Key Laboratory of Component-based Chinese Medicine, Tuanbo New Town, Tianjin University of Traditional Chinese Medicine, Tianjin, China; ^2^ Research and Development Center of TCM, Tianjin International Joint Academy of Biotechnology and Medicine, Tianjin Economic Developing Area, Tianjin, China

**Keywords:** ACT001, septic shock, sepsis-induced cardiomyopathy, cecal ligation and puncture, coagulation, JAK-STAT signaling pathway

## Abstract

Sepsis is a life-threatening multiple organ dysfunction syndrome (MODS) caused by a microbial infection that leads to high morbidity and mortality worldwide. Sepsis-induced cardiomyopathy (SIC) and coagulopathy promote the progression of adverse outcomes in sepsis. Here, we reported that ACT001, a modified compound of parthenolide, improved the survival of sepsis mice. In this work, we used cecal ligation and puncture (CLP) model to induce SIC. Transthoracic echocardiography and HE staining assays were adopted to evaluate the influence of ACT001 on sepsis-induced cardiac dysfunction. Our results showed that ACT001 significantly improved heart function and reduced SIC. Coagulation accelerates organ damage in sepsis. We found that ACT001 decreased blood clotting in the FeCl_3_-induced carotid artery thrombosis experiment. ACT001 also reduced the production of neutrophil extracellular traps (NETs). RNA-sequencing of heart tissues revealed that ACT001 significantly downregulated the expression of pro-inflammatory cytokines and the JAK-STAT signaling pathway. These results were confirmed with real-time PCR and ELISA. In summary, we found ACT001 rescued mice from septic shock by protecting the cardiovascular system. This was partially mediated by inhibiting pro-inflammatory cytokine production and down-regulating the JAK-STAT signaling.

## Graphical Abstract

**Figure:**
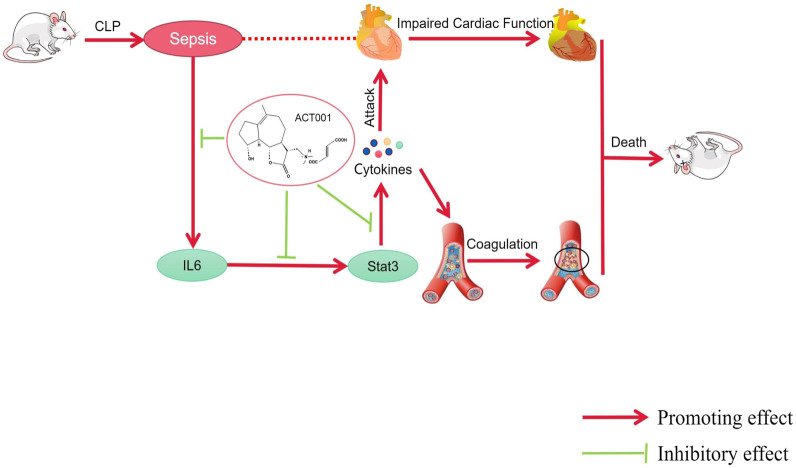
ACT001 improves cardiovascular function in sepsis mice by inhibiting the expression of IL-6, STAT3, and pro-inflammatory cytokines.

## Highlights

1. ACT001 rescued mice from septic shock and protected cardiac function during sepsis.
2.ACT001 alleviated sepsis-induced cardiac dysfunction by normalizing cytokine production and down-regulating the JAK-STAT signaling pathway.

## Introduction

Sepsis is defined as life-threatening organ dysfunction caused by the dysregulated host response to infection (Singer et al., 2016). The mortality rate of septic shock is higher than 40% (Napolitano, 2018). The treatment cost for sepsis is an enormous economic burden to society (Tiru et al., 2015). Nowadays, sepsis is one of the main challenges worldwide. Sepsis has complex mechanisms (Singer et al., 2016). Increased mortality in septic shock strongly correlates with myocardial dysfunction and disseminated intravascular coagulation (DIC) (Vincentet and De Backer, 2013). Targeting both myocardial dysfunction and coagulopathy may improve the prognosis of sepsis.

Sepsis is accompanied by a variety of complications along with its occurrence and development. DIC has been identified as one of the fatal complications of sepsis. In addition to excessive inflammatory response, the over-activated coagulation system causes hyper-coagulability, hypofibrinolysis, microthrombosis, and endothelial dysfunction, which trigger multiple organ failures. Results of clinical trials suggested that a lower level of activated partial thromboplastin time (aPTT) is associated with lower mortality in patients with septic shock (Massion et al., 2012). The activation of the coagulation system during sepsis strongly associates with organ dysfunction and hemorrhage (Iba et al., 2019; Iba et al., 2020). In addition, a recombinant human soluble thrombomodulin reduced mortality in patients with sepsis-induced DIC without increasing the bleeding risk during the treatment (Kato et al., 2023).

Sepsis-induced cardiomyopathy (SIC) is one of the most common complications of sepsis and is a chief manifestation of multiple organ failure, leading to high mortality (He et al., 2020; Hollenberg and Singer, 2021). Sepsis is associated with systemic inflammation (Huang et al., 2019). Pro-inflammatory factors, such as tumor necrosis factor α (TNF-α), interleukin 1β (IL-1β), interleukin 2 (IL-2), and interleukin 6 (IL-6) are signatures of systemic inflammation (Garbers C et al., 2018; Huang et al., 2019). IL-6/STAT3 signaling pathway plays a critical role in sepsis, modulating the inflammatory response and coagulation (Xu et al., 2020; Lei et al., 2021). Jahangiri found that inhibiting STAT3 could reduce the production of HIF-1α dependent immunosuppressive cytokines and thus enhance immunity (Wang et al., 2020).

Current therapeutic strategies for sepsis rely on suitable antibiotics and hormones to eradicate infection (Howell and Davis, 2017; Gavelli et al., 2021). Although therapeutic outcomes have improved over the past decades, antibiotic resistance and adverse reactions are the leading causes of adverse consequences (Strich et al., 2020). Therefore, searching for new effective drugs and therapeutic targets is urgent. ACT001 (also known as DMAMCL) showed anticancer activity and has been used to treat different cancers in clinical trials. It is certified as an orphan drug by the Food and Drug Administration in the United States. ACT001 (AC) was developed by modifying extracted parthenolide (PTL) ((Zhang et al., 2012; Zhang et al., 2012; Liu et al., 2020a; Zhu et al., 2021). PTL has anti-inflammatory and anticancer effects, but poor water solubility and stability limit its clinical application. The water-soluble ACT001 is produced on an industrial scale at a relatively low cost. It can cross the blood-brain barrier and reduce the expression of PD-L1 by inhibiting the phosphorylation of STAT3 in glioblastoma (Tong et al., 2020). It has significant anti-inflammatory and anti-platelet effects (Wang and Li, 2015). We wondered whether AC can reduce the mortality rate in sepsis by improving cardiovascular function dysfunction. Thus, we used the clinically relevant cecal ligation and puncture (CLP) model to address our questions (Rittirsch et al., 2009; Wang et al., 2021).

In this work, we found AC improved the survival and protected cardiovascular function of CLP mice. On the molecular level, it normalized proinflammatory cytokine production and downregulated the JAK-STAT signaling pathway in the cardiac tissue.

## Methods

### Chemicals and reagents

Xuebijing injection (XBJ) (batch number: 2108101) was produced by Tianjin Chase Sun Pharmaceutical Co., Ltd. (Tianjin, China). ACT001 was produced by Accendatech (Tianjin, China). 2, 2, 2-Tribromoethanol was produced by Sigma-Aldrich (Shanghai, China). Percoll Cell Separation Buffer (Cat. No. P8370) and Sterile Red Blood Cell Lysis Buffer (Cat.No. R1010), BCA protein concentration assay kit (PC0020), and SDS-PAGE gel preparation kit (P1200) were produced by Solarbio (Beijing, China). Phorbol-12-myristate-13-acetate (PMA) (Cat. No. HY-18739) was produced by MedChemExpress (Monmouth Junction, NJ, USA). Goat Anti-Rabbit IgG (H + L) HRP (S0001) was produced by Affinity (Melbourne, Australia).

### Experimental animals and ethical statement

The study was conducted following the recommendations of the Guide for the Care and Use of Laboratory Animals (NIH Publication No. 85–23, revised 1996, United States) and the recommendations in the Guidance for the Care and Use of Laboratory Animals issued by the Ministry of Science and Technology of China. All experiments were approved by the Experimental Animal Ethics Committee of Tianjin University of Traditional Chinese Medicine (Tianjin, China). We purchased 7-week-old male ICR mice from Beijing Vital River Laboratory Animal Technology Co., Ltd. (Beijing, China, Certificate number: SCXK Jing 2018–0011). The mice were kept in controlled temperatures (22 ± 2°C) and relative humidity (40% ± 5%), fed commercial mouse food and purified water, and had a 12-h light/dark cycle. Mice were used for experiments after 1 week of adaptive feeding.

### Cecal ligation and puncture

As described previously (Wang et al., 2021), the abdomen of anesthetized mice was depilated, and an incision of less than 1 cm was cut with scissors to expose the cecum which was ligated in 1/3 from the ileocecal valve with a 2–0 wire. We perforated the cecum with an 18G needle and then squeezed out a small amount of feces. After the operation, the cecum was returned to the original position, the wound was sutured with a 4–0 suture, and the mice were kept on an electric blanket after the operation.

### Drug administration

The experimental mice were randomly divided into Sham group, CLP group, CLP + ACT001 group and CLP + XBJ group. Pre-administration was performed by caudal vein injection the day before the CLP procedure. Two hours after CLP, the CLP + XBJ group and CLP + ACT001 group were injected with XBJ (9 mL/kg) and ACT001 (12.5 mg/kg), respectively, twice a day. The Sham group and the CLP group were injected with the same volume of saline. In the 7-day survival experiment, the mice were given XBJ or ACT001 until the seventh day.

### ELISA

Twenty-four hours after CLP, blood was collected and left at room temperature for 30 min. The clot was removed by centrifuging at 1,500 g, 4°C for 10 min in a centrifuge. The TNF-α and IL-6 were detected using ELISA kits with an automatic biochemical analyzer (Multiskan MK3; Thermo Fisher Scientific, Waltham, MA, United States) according to the manufacturer’s instructions as previously described (Lyu et al., 2018a; Chen et al., 2018).

### Hematoxylin and eosin staining

Hematoxylin and eosin (HE) staining was described previously (Xiao et al., 2019). In short, 24 h after CLP surgery, cardiac tissue was removed from dissected mice and fixed in 4% formaldehyde solution at room temperature for at least 48 h. Next, programmed dehydration and paraffin embedding were performed. Then, the tissue blocks were sectioned with a manual microtome to obtain 4 μm-thick sections, and finally, HE staining was performed at room temperature. The pictures were taken with an Olympus microscope. As described previously (Wen et al., 2020), scoring standard (presence of inflammatory cells) in H&E stained tissue sections was scored as 0 (absent), 1 (focal/mild, ≤1 foci), 2 (moderate, ≥2 inflammatory foci), 3 (extensive coalescing of inflammatory foci or disseminated inflammation), and 4 (diffuse inflammation, tissue necrosis, interstitial edema, and loss of integrity).

### Transthoracic echocardiography in mice

As described previously (Wang et al., 2021), 24 hours after CLP surgery, transthoracic echocardiography was performed on the aorta and the left ventricle of the heart in mice using Vevo 2100 Imaging System (Visual Sonics, Toronto, ON, Canada) to determine the cardiac function of mice. The animals were removed from the induction chamber and the hair on the chest was removed with a depilatory cream. The anesthetized mice were lying on a heating pad with embedded ECG leads to maintain body temperature. Nose cone connected to the anesthesia system was used to maintain a stable sedation level throughout the process (1.0%–1.5% isoflurane mixed with 0.5 L/min of 100% O_2_). The level of anesthesia was adjusted to achieve a target heart rate (bpm) of 450 ± 50 beats per minute. Four claws were attached to the ECG electrode with electrode gel. The probe was gently placed on the mouse’s chest to locate the left ventricle during the testing. Three cardiac cycles were measured for each mouse and the average value was taken. All data were analyzed after the experiment using the software provided with the ultrasound system.

### FeCl_3_ induced thrombosis

As described (Li et al., 2016), FeCl_3_ thrombus model was used to evaluate the anticoagulant effect of the drug *in vivo*. Eight-week-old ICR mice were divided into Control group, ACT001 group, XBJ group, and heparin sodium group. The heparin sodium concentration (63 U/mL) was converted according to the clinical dose conversion formula, and each mouse was injected with 0.25 mL.

ICR mice were treated with ACT001, XBJ, or heparin 4 days before the experiment. All treatments were administered through the tail vein injection twice a day. Four days after administration, the mice were subjected to FeCl_3_ -induced thrombus procedure and imaging as described (Li et al., 2016). The blood clotting was recorded for 30 min for each mouse. The time of thrombosis formation and the final blood flow value of each group were compared to evaluate the anticoagulant effect of ACT001.

### Tail bleeding and coagulation experiment

Tail bleeding experiment (Carol Illa et al., 2021; Østergaard et al., 2021): Prepare a 100 mL beaker, saline preheated to 37°C, a white background plate, and a high foam brick. Pour the preheated saline into the beaker, put a background plate between the beaker and the foam brick, and put an electric mat on the bottom of the beaker to keep the temperature of the saline. After anesthesia, the tail tip was cut off at a distance of 3 mm from the tail tip and quickly placed on the foam brick. The tail was immersed in the beaker to start timing, observe the bleeding situation, and stop timing when the blood flow stopped.

Tail clotting experiment (Wu et al., 2015; Bian et al., 2022): Prepare a clean petri dish and slide, wet the inside of the petri dish with warm water, put the clean slide in the dish, close the lid to keep the internal air moist, cut off the tail tip of the mouse to make it bleed naturally, start the stopwatch when the blood flows out, immediately close the lid when the blood drops onto the slide, open the lid every 15 s, insert the needle into the blood drop and lift it. If fibrin filaments were observed, the stopwatch was paused and the bleeding time was recorded.

### Visualization of neutrophil extracellular traps production

The formation of NETs was induced according to the method in the literature (Stoikou et al., 2017; Hawez et al., 2019). The obtained neutrophils were seeded into a 96-well plate at a density of 10,000 per well and allowed to stand for 4 h until adherence. The cells were incubated with ACT001 (30 μM) and XBJ (1:50 dilution), simultaneously 0.5 μM PMA was used to stimulate neutrophils to form NETs, and then fixed the cells for immunofluorescence experiments. The operation process is as follows: Fixing cells with 4% paraformaldehyde at room temperature for 30 min; 0.5% Trition was added to the cells for 10 min at room temperature; the cells were blocked with 5% BSA at room temperature for 1 h. Then the cells were stained with anti-neutrophil elastase (1:200, bs-23549R, Bioss) and anti-citrullinated histone H3 (1:200, AF0863, Affinity) primary antibodies diluted in blocking buffer overnight at 4°C, and AlexaFluor 488-conjugated IgG antibody (1:500; Abcam) was stained for 2 h in the dark at room temperature. Hoechst 33342 (catalog no. H1399, Thermo Fisher Scientific, Waltham, MA, United States) at 1 mg/mL was used to stain the nucleus. Images were acquired with a PerkinElmer high-content imaging system as described (Wang et al, 2021).

### RNA samples collection

As described (Wang et al., 2021), 24 hours after CLP surgery, the heart tissues of mice were collected. We washed the blood from the intracavity with normal saline and immediately placed the hearts in liquid nitrogen. The hearts were later used for high-throughput sequencing on an Illumina sequencing platform (n = 4 in each group). RNA was extracted from cardiac tissues using the standard extraction method as described (Lyu et al., 2018b) and was reverse transcribed using the NEB Next®Ultra™RNA library preparation kit for Illumina^®^.

### RNA-sequencing

The obtained cardiac tissue RNA was extracted to construct a gene library and then subjected to Illumina high-throughput sequencing. The poly-N, adapter and low-quality reads contained in the original data were deleted to obtain clean reads to ensure the quality and reliability of downstream data analysis; then the comparison software HISAT2 (The Johns Hopkins University, Baltimore, Maryland, United States) was used to compare The obtained Clean Reads were compared with mouse genes, and the number of reads of each gene was calculated according to the location of the reference genome; the Feature Counts tool of Subread software was used to quantify the gene expression level; DESeq2 software was used to analyze the differential expression between groups, a corrected *p*-value of 0.05 and an absolute fold change of two were set as the threshold for significantly differential expression.

### GO and KEGG enrichment analysis of differentially expressed genes

Gene Ontology (GO) enrichment analysis of differentially expressed genes was implemented using the cluster Profiler R package, in which gene length bias were corrected. GO terms with corrected *p*-value less than 0.05 were considered significantly enriched by differentially expressed genes. KEGG is a database resource for understanding high-level functions and utilities of the biological system. We used the cluster Profiler R package to test the statistical enrichment of differential expression genes in KEGG pathways.

### Real-time PCR

Real-time PCR experiments were performed as previously described (Shang et al., 2022). The total RNA was extracted using Trizol, including lysing cells, isolating, precipitating, and washing RNA. The total RNA was dissolved in ddH2O. All cDNA was prepared following the protocol of Transcriptor First Strand cDNA Synthesis Kit (Cat#: 04896866001, Roche Life Science). The relative mRNA level was determined using the comparative CT method and was normalized to the housekeeping gene glyceraldehyde-3-phosphate dehydrogenase (GAPDH). The primers were synthesized by Sangon Company (Shanghai, China). The primer sequences for real-time PCR were presented in Table 1.

**TABLE 1:** The primer sequences for real-time PCR experiments.

Gene	Forward primer (5'→3′)	Reverse primer (5'→3′)
GAPDH	GGT​TGT​CTC​CTG​CGA​CTT​CA	TGG​TCC​AGG​GTT​TCT​TAC​TCC
JAK3	CCA​TCA​CGT​TAG​ACT​TTG​CCA	GGC​GGA​GAA​TAT​AGG​TGC​CTG
CSF3	ATG​GCT​CAA​CTT​TCT​GCC​CAG	CTG​ACA​GTG​ACC​AGG​GGA​AC
IL-6	TAG​TCC​TTC​CTA​CCC​CAA​TTT​CC	TTG​GTC​CTT​AGC​CAC​TCC​TTC
SOCS3	ATG​GTC​ACC​CAC​AGC​AAG​TTT	TCC​AGT​AGA​ATC​CGC​TCT​CCT
STAT3	AGC​TGG​ACA​CAC​GCT​ACC​T	AGG​AAT​CGG​CTA​TAT​TGC​TGG​T

### Statistical analysis

All tests were performed using GraphPad Prism eight software (GraphPad Software, Inc., La Jolla, CA, United States). All data were expressed as the mean ± SEM or mean ± SD. Statistical analysis was carried out using Student’s two-tailed *t*-test for comparison between two groups. One-way analysis of variance (ANOVA) followed by Dunnett’s test was used to analyze the data involving three or more groups. *p* < 0.05 was considered statistically significant.

## Results

### ACT001 improved survival and reduced the sepsis-induced histopathological damage in the cardiac tissues

**FIGURE 1:**
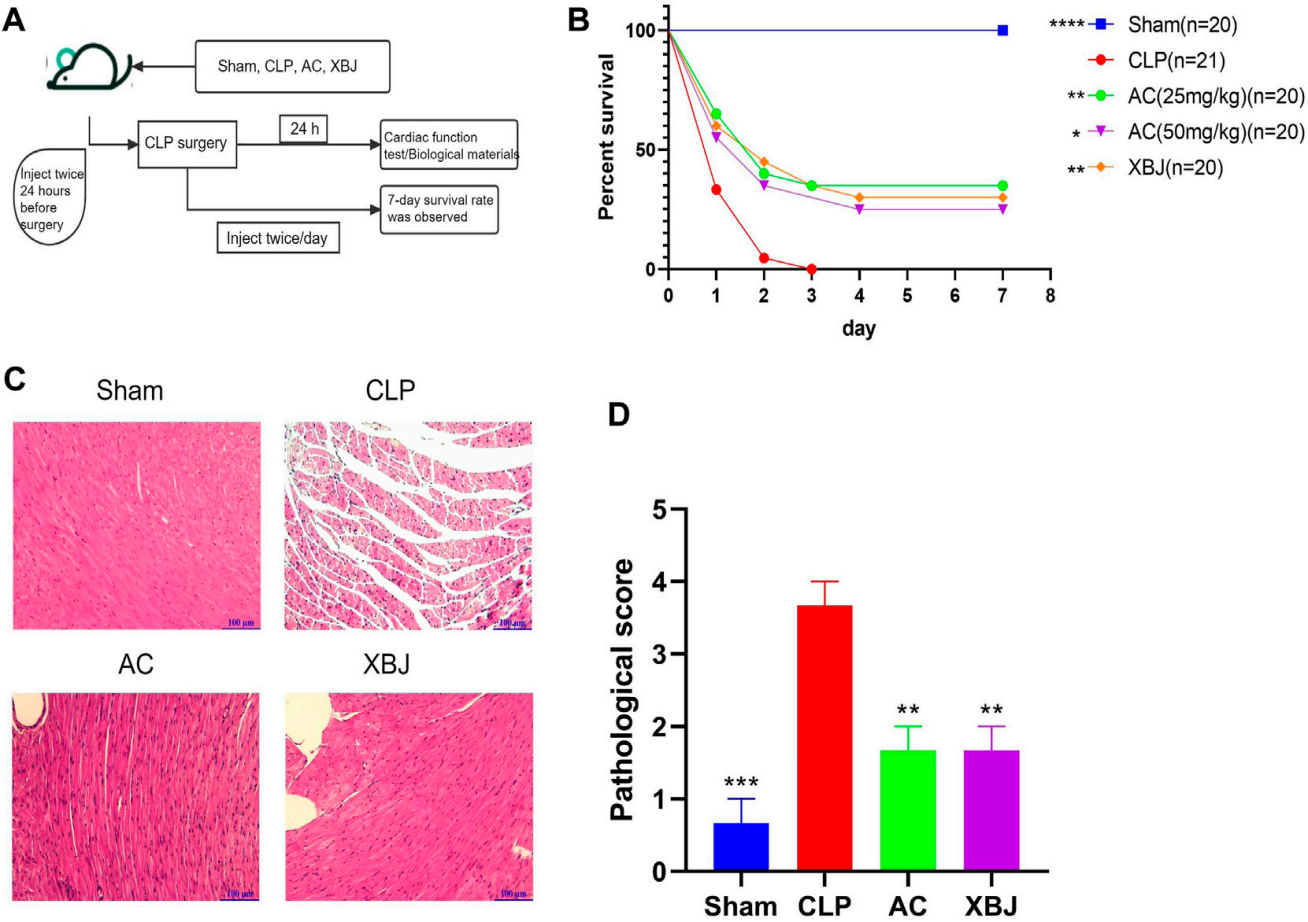
ACT001 improved survival of sepsis mice and reduced the sepsis-induced histopathological damage in the cardiac tissues. **(A)** Schematic diagram of ACT001 treatment schedule and the experimental plan; **(B)** The 7-day survival curve of sepsis mice and the treatment groups (n ≥ 20); **(C)** HE staining of representative heart tissue sections of the Sham, CLP, ACT001-treated, and XBJ-treated mice. **(D)** Pathological score of cardiac staining (n = 3). Results were presented as mean ± SEM. *p < 0.05, **p < 0.01, ***p < 0.001, ****p < 0.0001, vs. CLP group.

We designed a series of experiments to determine the influence of ACT001 on CLP mice *in vivo* (Figure 1A). We evaluated the therapeutic effect of different doses of ACT001 in CLP mice. ACT001 at the dosage of 25 mg/kg/day significantly improved the survival of septic mice (Figure 1B). Therefore, this dose was selected for subsequent experiments. Cardiac injury and dysfunction are common complications of sepsis, HE staining was used to measure the heart tissue damage at 24 h after CLP. As shown in Figure 1C, abnormal myocardial morphology, such as myocardial rupture and separation, was observed in CLP mice. ACT001 treatment protected the integrity of cardiac structure in septic mice.

### ACT001 protected cardiac function in sepsis mice

**FIGURE 2:**
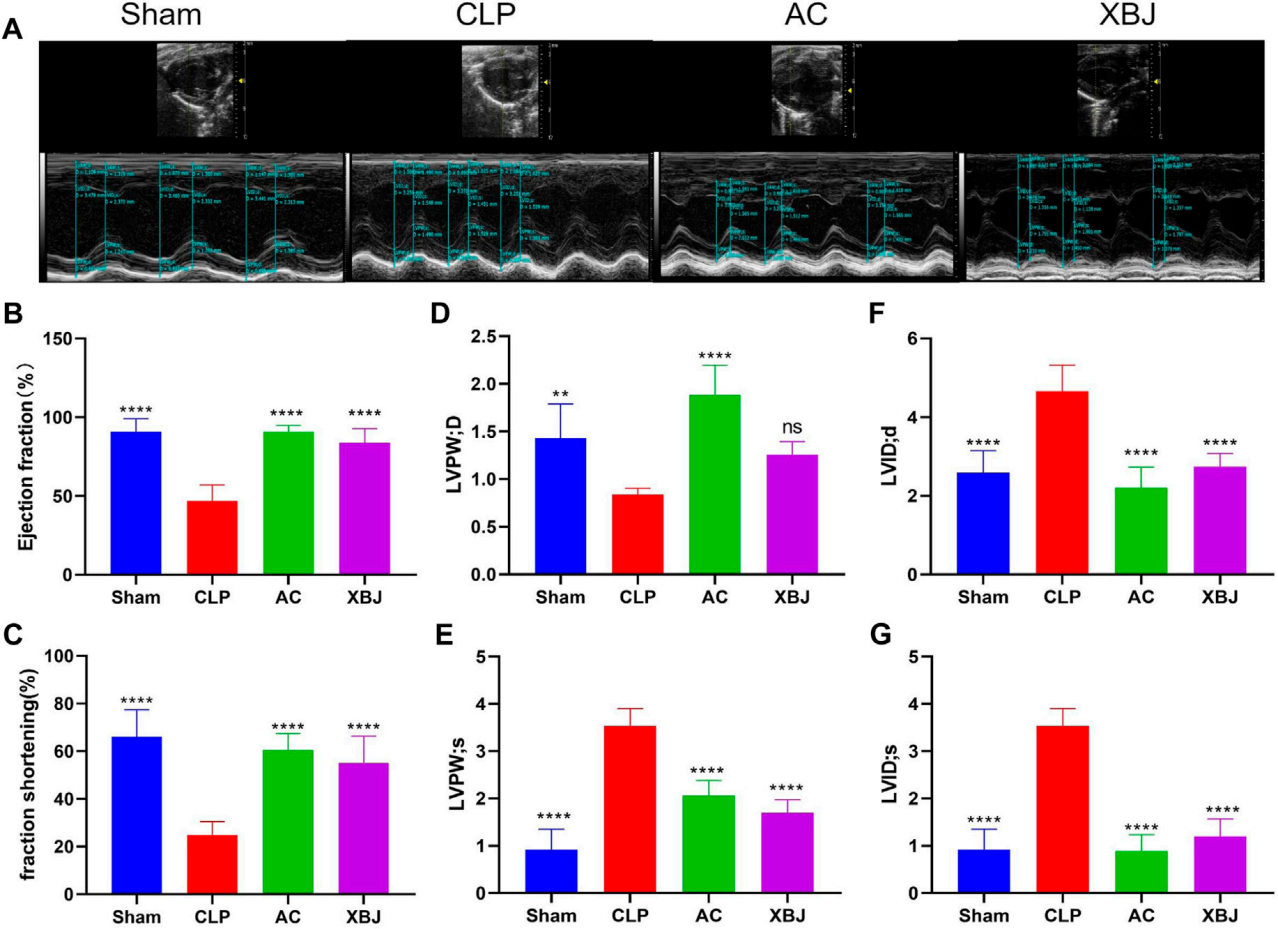
ACT001 improved the cardiac function of sepsis mice. **(A)** Cardiac performance was determined by echocardiography in different groups as indicated. Representative images of left ventricular echocardiography of each group of mice were presented. Left ventricular ejection fraction (LVEF) % **(B)** and left ventricular fractional shortening (LVFS) % **(C)** were measured in M-mode. LV posterior wall diastole (LVPWd) **(D)**, LV posterior wall systole (LVPWs) **(E)**, left ventricular internal dimensions at diastole (LVIDd) **(F)**, and left ventricular internal diameter systole (LVIDs) **(G)** were also measured and quantified. Results were presented as mean ± SEM (n = 6–7). *p < 0.05, **p < 0.01, ***p < 0.001, ****p < 0.0001, vs. CLP group.

Cardiac function was evaluated at 24 h after CLP to determine the influence of ACT001 (Zhai and Guo, 2016). Figure 2A exhibited representative images of echocardiography in all groups. CLP mice displayed detrimental cardiac function, including reduced left ventricular ejection fraction (LVEF%), left ventricular fractional shortening (LVFS%) and left ventricular posterior wall diastole (LVPWd). While left ventricular internal diameter diastole (LVIDd), Left ventricular posterior wall end systole (LVPWs), and left ventricular internal diameter systole (LVIDs) were increased compared with the Sham mice (Figures 2B–G). However, ACT001 significantly improved the cardiac function of septic mice (Figures 2B–G).

### ACT001 delayed the FeCl_3_-induced thrombosis *in vivo*

**FIGURE 3:**
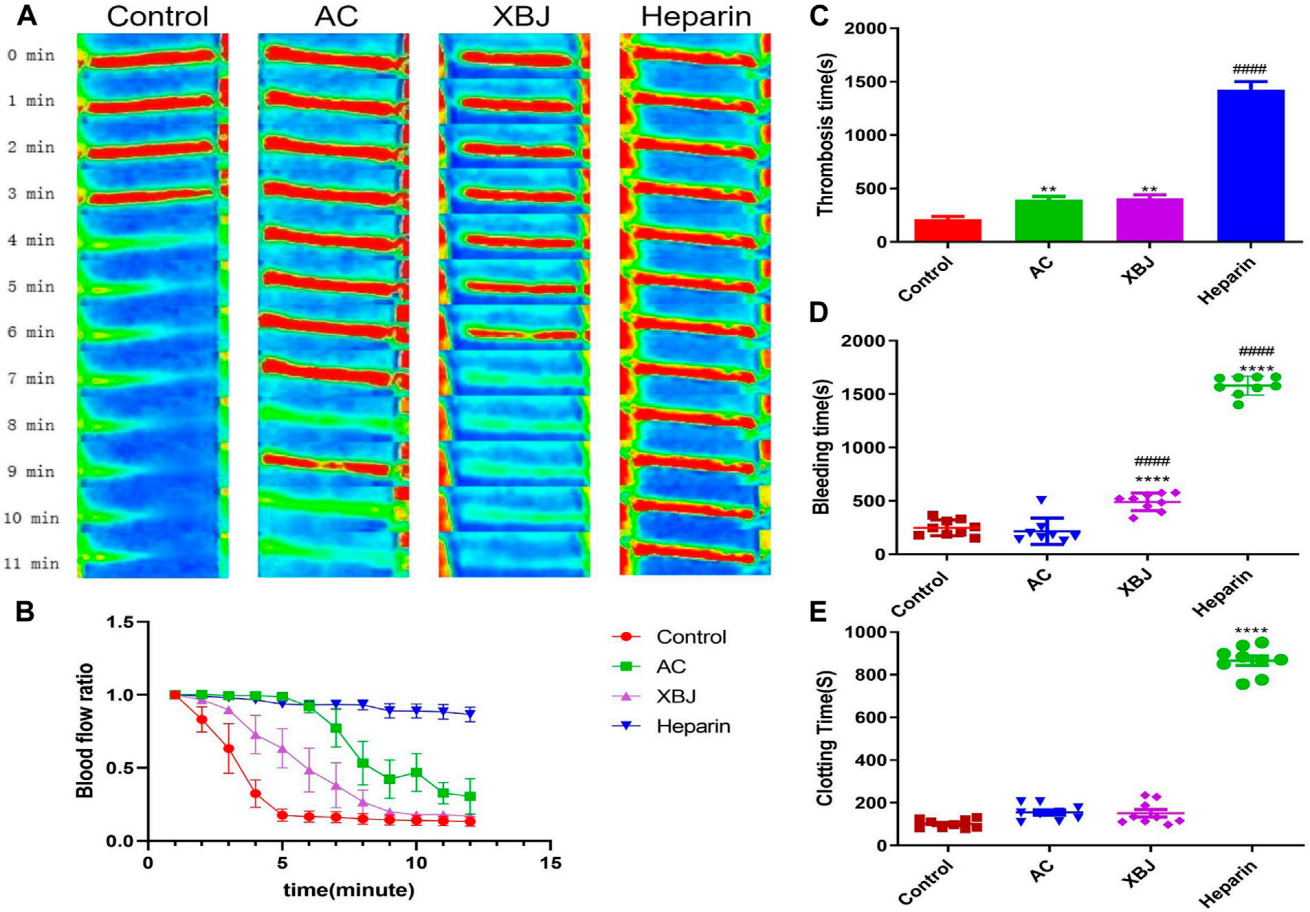
ACT001 delayed thrombosis in mice. **(A)** The representative blood flow imaging of the carotid artery under FeCl_3_ stimulation in each group of mice. **(B)** Changes in blood flow ratio after carotid FeCl_3_ stimulation at different time points in each group. The unit of time is minute. **(C)** Thrombosis time after carotid FeCl_3_ stimulation in each group. **(D)** Comparison of bleeding time after tail tip truncation in mice treated with different drugs. **(E)** Comparison of tail-tip blood clotting time in mice after different drug treatments. Results were expressed as mean ± SEM (n = 4–9). *p < 0.05, **p < 0.01, ****p < 0.0001, vs. control group.

Sepsis-induced disseminated intravascular coagulation (DIC) threatens the life of sepsis patients (Giustozzi et al., 2021). We evaluated the anti-thrombotic effects of ACT001 in the FeCl_3_-induced arterial thrombosis mouse model. As shown in Figure 3A, FeCl_3_ damaged endothelial cells and triggered thrombus formation in the carotid artery. ACT001 treatment significantly prolonged the coagulation time (Figures 3B,C). To further verify the safety of ACT001 on coagulation in healthy mice, tail hemorrhage and coagulation experiments were conducted. ACT001-treated mice didn’t show a significant difference in bleeding time (Figure 3D) and clotting time (Figure 3E) compared with the control group. The above results indicated that ACT001 has the potential to prevent and treat sepsis-induced coagulopathy.

### ACT001 reduced the neutrophil extracellular trap formation

**FIGURE 4:**
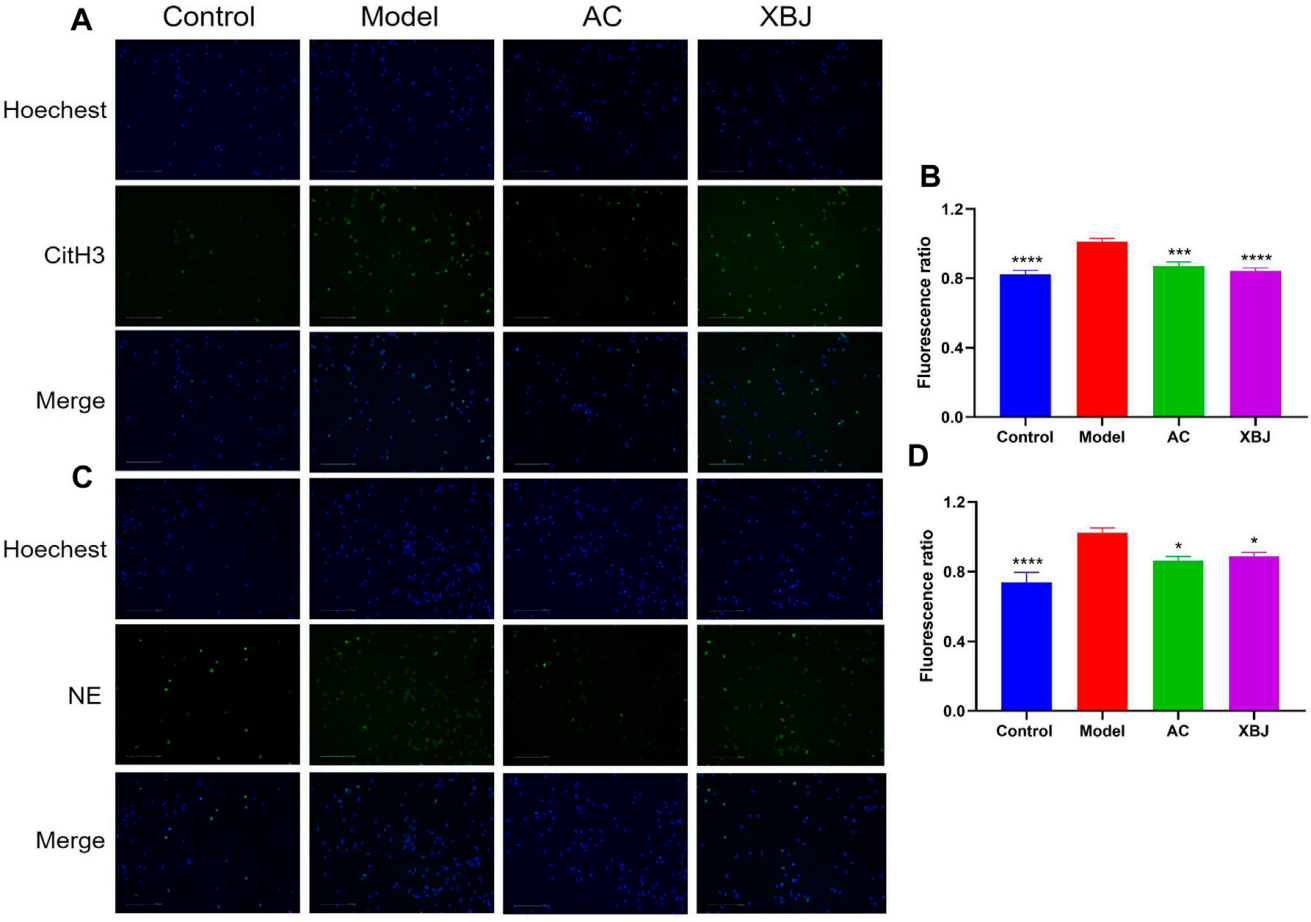
ACT001 inhibited neutrophil extracellular traps (NETs) formation. The purified neutrophils from bone marrow were stimulated with PMA and incubated with/without ACT001. **(A,B)** NETs formation was detected by immunofluorescence staining for CitH3 (green) and Hoechst (blue). **(C,D)** NE expression level was determined by immunofluorescence assay to reveal the NETs formation. The experiment was repeated for three times. Results were presented as mean ± SEM. *p < 0.05, ***p < 0.001, ****p < 0.0001, vs. Model group.

In sepsis, neutrophils play an essential role in the body’s innate immunity. Excessive activation of neutrophils induces NETs (Sollberger et al., 2018). NETs contribute to immunothrombosis and multiple organ dysfunction syndrome (MODS) (McDonald et al., 2017; Abrams et al., 2019; Frangou et al., 2019). We isolated neutrophils from bone marrow and evaluated the efficacy of ACT001 on Phorbol-12-myristate-13-acetate (PMA)-induced NETs formation. Since 30 μM ACT001 did not cause cytotoxicity in the CCK-8 cell viability test, we tested the influence of 30 μM ACT001 on NETs formation *in vitro*. In the immunofluorescence assay, the expressions of citrullinated histone H3 (CitH3) and neutrophil elastase (NE) were induced by PMA. In contrast, ACT001 significantly reduced their expression levels compared with the model group (Figure 4).

### ACT001 normalized the gene expression profile in the cardiac tissue of sepsis mice

**FIGURE 5:**
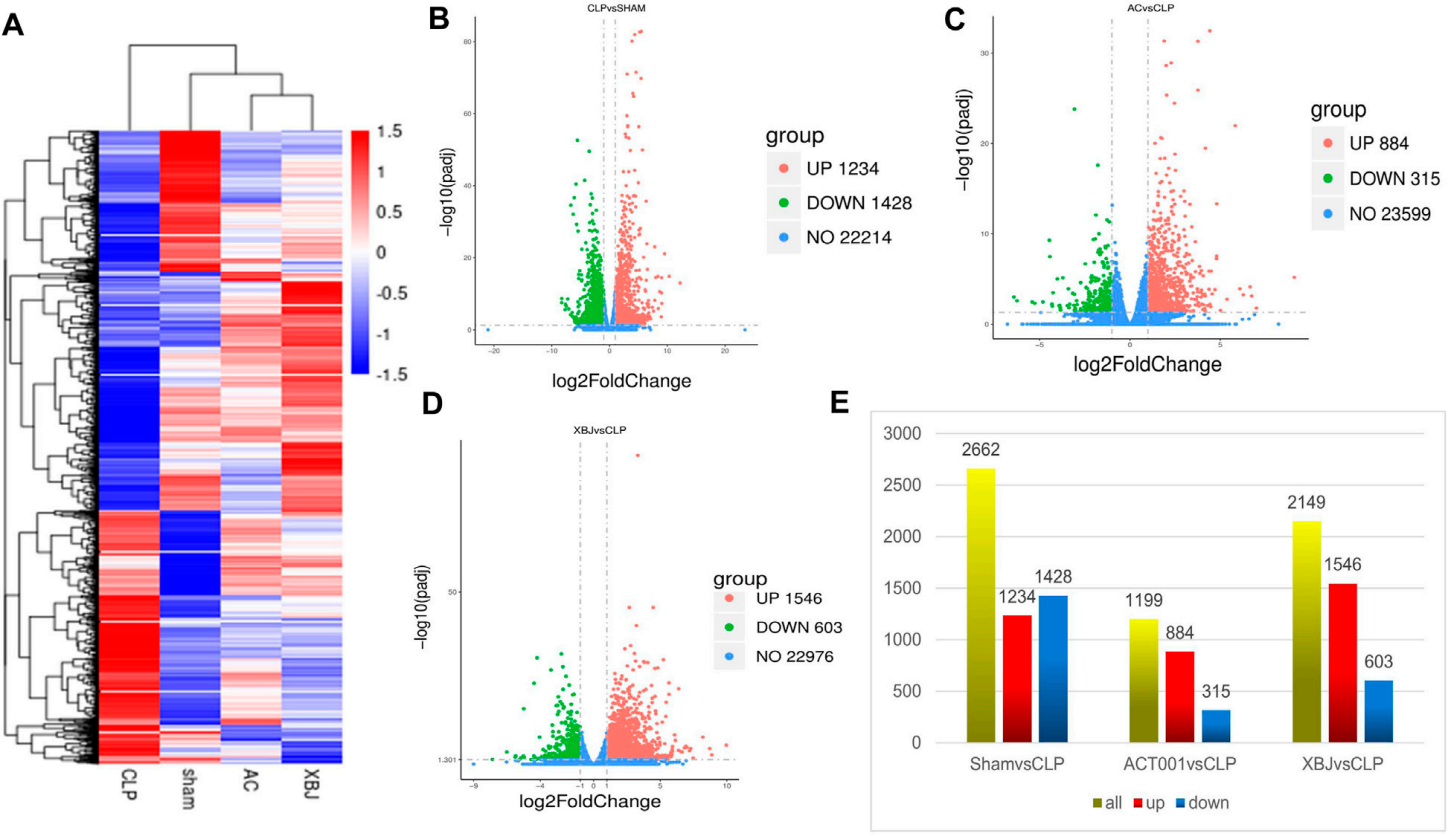
ACT001 normalized the gene expression profile in the cardiac tissues of sepsis mice. 24 h after CLP, the cardiac tissues of different groups were harvested and subjected to RNA-seq analysis. **(A)** Heat map of the gene expression profiles in different groups. **(B–D)** The overall distribution of differentially expressed genes (DEGs) (log2foldchange ≥1 and padj ≤0.05) in different groups was reflected by the volcanic map. The *X*-axis represented the changes of gene expression in different samples and *Y*-axis represented the statistical significance of the difference in the gene expression. The upregulated genes were marked in red and the downregulated genes were marked in green. **(E)** Differential gene counts between different groups.

We conducted RNA sequencing to determine the impacts of ACT001 on the gene expression profile of the cardiac tissue in septic mice. Figure 5A showed both ACT001 and XBJ impacted the overall gene expression profiles in sepsis mice. Figures 5B–E revealed the differentially expressed genes (DEGs) counts between different groups. There were 1199 DEGs in the ACT001 treatment group and 2149 DEGs in the XBJ treatment group with Log2FC > 1 and *p*-value<0.05 when compared with sepsis mice.

**FIGURE 6:**
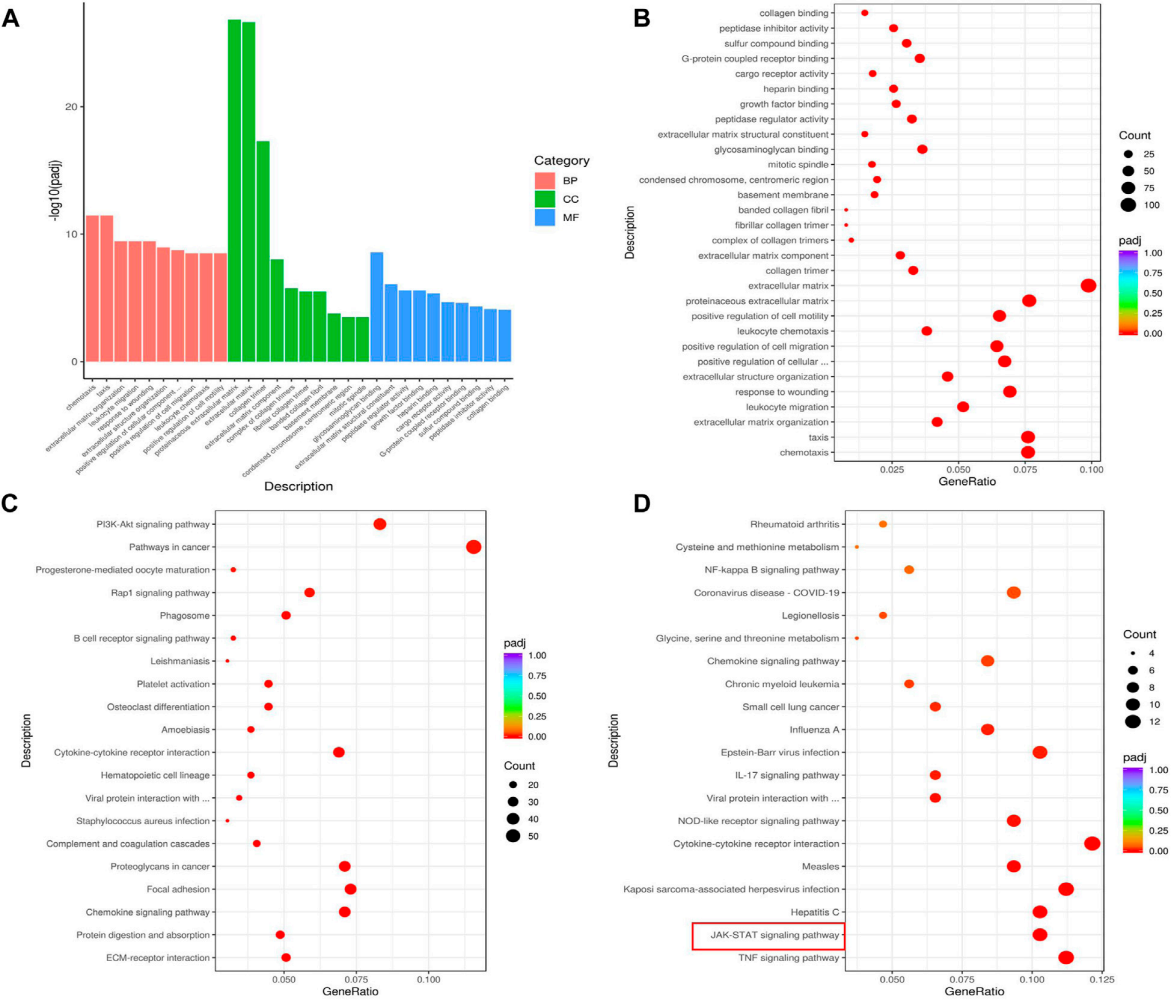
ACT001 influenced critical functions and pathways in sepsis mice. **(A,B)** GO analysis of the top 30 functions impacted by ACT001. BP, biological process; CC, cellular component; MF, molecular function. **(C)** KEGG analysis of top 20 pathways of all DEGs impacted by ACT001. **(D)** The top 20 signaling pathways of all downregulated DEGs regulated by ACT001 were shown. The pathways/functions were ranked from left to right by −log(padj adjusted).

We further analyzed DEGs using GO and KEGG analysis to reveal the signaling pathways regulated by ACT001. As shown in Figures 6A,B, chemotaxis, taxis, extracellular matrix organization, leukocyte migration, and response to wounding are among the top 10 biological processes regulated by ACT001. The top 10 signaling pathways regulated by ACT001 include cytokine-cytokine receptor binding, viral protein interaction with cytokine and cytokine receptor, MAPK signaling pathway, and NF-kappa B signaling pathway (Figure 6C). To understand the working mechanism of ACT001 on sepsis-induced cardiac dysfunction, we analyzed the 315 downregulated genes. The top 2 downregulated signaling pathways were the TNF signaling and the JAK-STAT signaling (Figure 6D).

#### ACT001 suppressed the JAK-STAT signaling pathway in the cardiac tissue of sepsis mice

**FIGURE 7:**
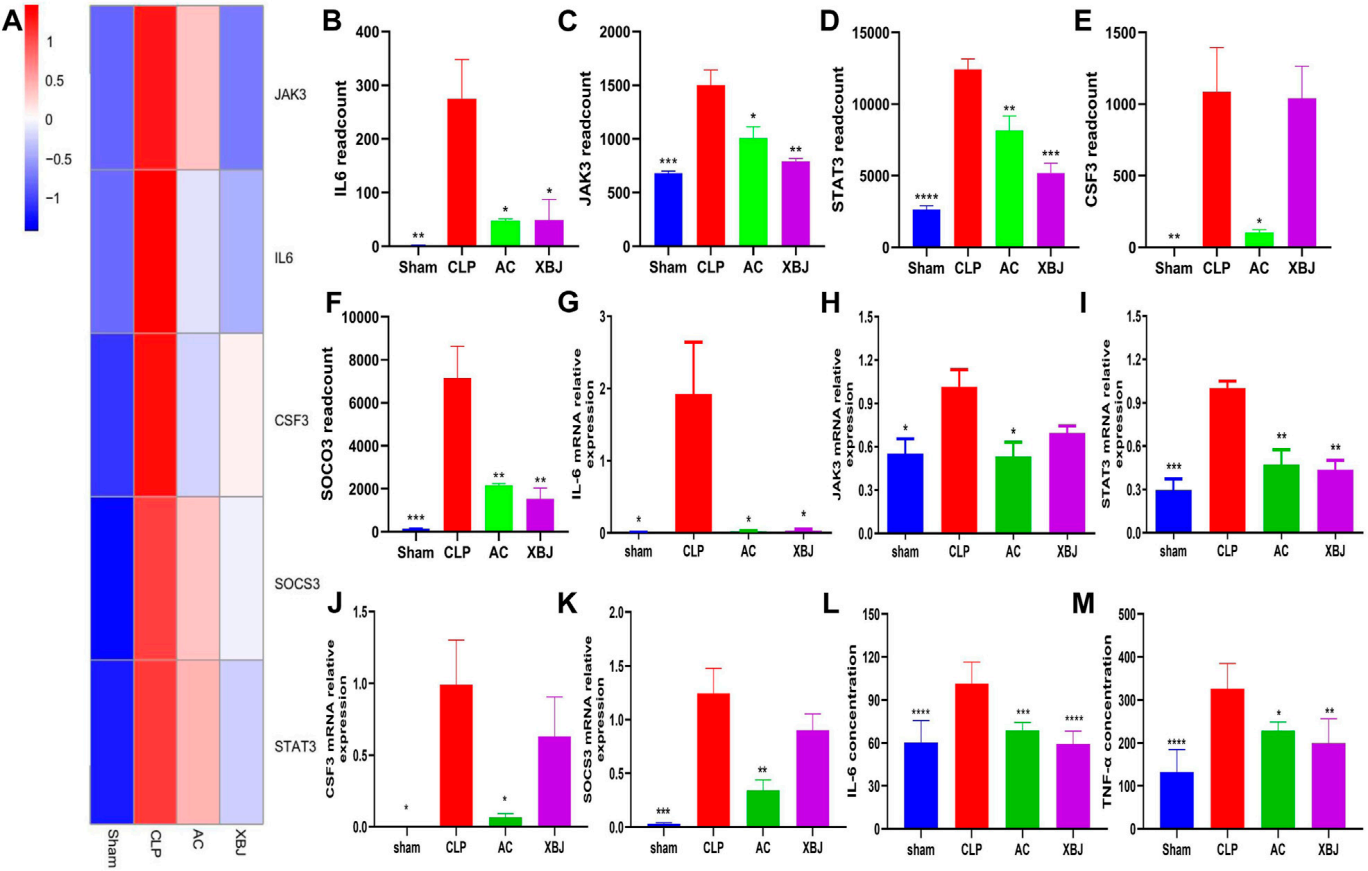
ACT001 reduced the gene expressions of JAK-STAT signaling pathway in the cardiac tissue of sepsis mice. **(A)** Heat map of the gene expression influenced by AC and XBJ in JAK-STAT signaling. **(B–F)** The expression of IL-6 **(B)**, JAK3 **(C)**, STAT3 **(D)**, CSF3 **(E)** and SOCS3 **(F)** were detected in the cardiac tissue of different groups of mice. Real-time PCR and ELISA were conducted to confirm the results of RNA-seq. (**G-M**) The relative mRNA expression levels in JAK-STAT signaling pathway including IL-6 **(G)**, JAK3 **(H)**, STAT3 **(I)**, CSF3 **(J)** and SOCS3 **(K)** were quantified with Real-time PCR. **(L,M)** The levels of IL-6 **(L)** and TNF-α **(M)** in serum were measured using the enzyme-linked immunosorbent assay (ELISA). All experiments were repeated for three times. Each bar was represented as mean ± SD (n = 5). *p < 0.05, **p < 0.01, ***p < 0.001, vs. CLP group.

Given the key role of the JAK-STAT signaling pathway in sepsis, we further validated the genes in the JAK-STAT signaling pathway. Real-time PCR was used to verify the accuracy of transcriptome results. Five verified genes were IL-6, JAK3, STAT3, CSF3, and SOCS3, respectively (Figures 7G–K). The results of Real-time PCR were consistent with the RNA-seq results (Figures 7A–F). ACT001 inhibited the expressions of these genes, suggesting that the transcriptome data were reliable and repeatable. Inflammatory cytokines play a critical role in septic progress. ELISA results showed that IL-6 and TNF-α levels in serum were significantly upregulated in the CLP group compared with the Sham mice, while ACT001 normalized their expressions on the protein level (Figures 7L,M).

## Discussion

We found ACT001 improved the survival of septic mice in our *in vivo* screening for a novel agent to manage sepsis. We were surprised that ACT001 showed a similar effect as our positive control, XBJ injection, in a series of *in vivo* and *in vitro* assays. We provided evidence in this study suggesting that ACT001 may halt the progression of septic shock by restoring homeostasis of the circulation system by targeting IL6/Stat3 signaling. This discovery may shed light on developing novel regimens to manage sepsis.

High mortality and morbidity rates in septic shock patients revealed limitations of the current sepsis treatments. There is a need for novel agents to restrain the threat of antibiotic-resistant bacteria. However, few agents are effective in managing antibiotic-resistant bacteria-related infections currently. Anti-inflammation and anti-infection characteristics of MCL, the active metabolite of ACT001, provided a rationale to test its influence on the clinically relevant CLP model (Qin et al., 2016; Jiang et al., 2017).

### ACT001 improved the survival of cecal ligation and puncture mice

ACT001 improved the survival of CLP mice (Figure 1B). Similar to XBJ treatment, 25 mg/kg/d ACT001 (twice/day) improved the survival of CLP mice to 35%. The t_max_ value of ACT001 in most tissues of rats is about 0.5 h. After 3 hours, the concentration of ACT001 in the tissues decreased significantly. At 10 h, the drug concentration dropped to a lower level (Xi et al., 2019). Therefore, we chose to administer ACT001 twice a day. In contrast, Qin et al. showed that 20 mg/kg MCL improved the survival of LPS-treated mice from 10% to 90% (Qin et al., 2016). Jiang et al., 2017 showed MCL improved the survival rate from 10% to 60% in an S. aureus-induced acute peritonitis model. ACT001 releases MCL *in vivo* (Zhang et al., 2012), indicating ACT001-derived MCL may play a role in managing systemic infection.

Previous results suggested that ACT001 may prevent organ failure in the CLP model to improve mice survival. Studies from different research groups revealed that ACT001 and MCL render organ protection in different disease models. Liu et al., 2019 found that ACT001 ameliorates diabetic kidney disease by inhibiting the expression of MTDH, an oncogene, in a murine diabetes model. In another study, Zhong et al., 2018 revealed a therapeutic effect of ACT001 on hepatic steatosis in a murine diabetes model. Wu et al. found that ACT001 protected the intestine in an irradiation-induced intestinal injury model (Wu et al., 2022). These results indicated that ACT001 may prevent sepsis-induced organ failure.

### The influence of ACT001 on the circulation system of sepsis mice

Dysfunctions of the circulation system, including sepsis-induced DIC and sepsis-induced myocardiopathy, are life-threatening complications (Iba and Levy, 2020; Giustozzi et al., 2021). They compromise the homeostasis of the circulation system during sepsis. So far, the influence of ACT001 on the circulation system during sepsis is not known. MCL attenuates cytotoxic drug-induced cardiotoxicity (Kalantary-Charvadeh et al., 2019; Yarmohammadi et al., 2021). It is believed that the protective actions of MCL against DOX-induced cardiotoxicity in mice are via repressing the PI3K/Akt/NF-kB signaling pathway (Kalantary-Charvadeh et al., 2019). These results inspired us to determine the effect of ACT001 on the cardiac function of sepsis mice. First, we determined the influence of ACT001 on cardiac function *in vivo*. ACT001 improved the morphology as well as the function of the heart in sepsis mice (Figures 1, 2), indicating a potential role of MCL in rendering cardiac protection. However, whether MCL has an equivalent impact as ACT001 on the cardiac function and gene expression profile remains to be determined.

### Influence of ACT001 on inflammatory cytokines

The crosstalk between inflammation and coagulation is closely linked to sepsis progression. Coagulation abnormalities are common in sepsis patients (Giustozzi et al., 2021). IL-6 is a trigger of sepsis-related coagulopathy. We found that ACT001 treatment reduced IL-6 expression on mRNA and protein levels in sepsis mice (Figure 7). This is consistent with results from other disease models (Patel et al., 2019; Zhang et al., 2022). It also decreased TNF-α in serum, suggesting ACT001 may ameliorate cytokine storm in sepsis mice.

### The influence of ACT001 on sepsis-associated coagulopathy

We did not retrieve reports about the influence of ACT001 or MCL on blood vessels and Thrombosis on Pubmed. However, PTL was reported to suppress thrombosis by inhibiting NF-kB signaling (Groenewegen and Heptinstall, 1990). In addition, either PTL-derived NF-kB inhibitors or unrelated NF-kB inhibitors can inhibit platelet aggregation (Malaver et al., 2009). Interestingly, both ACT001 and MCL attenuate NF-kB signaling which activates platelet and thrombosis (Kalantary-Charvadeh et al., 2019; Yarmohammadi et al., 2021), indicating ACT001 may hinder DIC/blood clotting in sepsis mice. As we predicted, ACT001 inhibited FeCl_3_-induced thrombosis *in vivo* (Figure 3). NETs formation triggers DIC in sepsis (Mao et al., 2021). In our *in vitro* NETs formation assay, ACT001 hindered NETs formation without compromising normal clotting (Figure 4), supporting its role in preventing pathogenic clotting. Overall, whether ACT001 prevents sepsis-induced coagulopathy by inhibiting NF-kB signaling remains to be studied.

### Anti-sepsis effect of ACT001

ACT001 turns into MCL *in vivo* (Zhang et al., 2012; Xi et al., 2019; Li et al., 2019). Previous studies revealed the therapeutic effect of MCL on LPS-induced septic shock and *Staphylococcus aureus* (SA) induced septic shock (Qin et al., 2016; Jiang et al., 2017). It is unclear whether MCL influences IL6/Stat3 signaling. Comparing the influence of ACT001 and MCL on septic shock in the future may reveal the potential working mechanism of ACT001 in managing septic shock.

### ACT001 may regulate IL-6 signaling to relieve sepsis-induced cardiac dysfunction

Activation of IL-6 signaling is a signature of sepsis. IL-6 is a therapeutic target of cytokine release syndrome and sepsis (Kang et al., 2020). It induces plasminogen activator inhibitor-1(PAI-1) in endothelial cells. Elevated PAI-1 contributes to the development of cardiovascular diseases (Sillen and Declerck, 2021). Our RNA-seq results revealed that ACT001 regulates IL-6/Stat3 signaling in the murine CLP model. It normalizes the expression of TNF, IL-6, JAK3, SOCS-3, and Stat3 at the mRNA level. These results were confirmed with Real-time PCR (Figure 7). The impact of ACT001 on IL-6 and TNF was further confirmed on protein level.

Consistent with our results, Jaffar et al. showed that ACT001 inhibits the expression of IL-6 in cells derived from pulmonary fibrosis (Jaffar et al., 2021). It inhibits the phosphorylation of Stat3 in glioblastoma (Tong et al., 2020). Given that IL-6 is an established therapeutic target (Martin et al., 2019; Kang et al., 2020) and Stat3 is an emerging target in managing sepsis (Hui et al., 2009; Hou et al., 2021; Lei et al., 2021), this is important for understanding the working mechanism of ACT001 in sepsis. Coincidentally, a recent publication has shown that XBJ can improve sepsis-induced myocardial damage and inflammation by regulating the NF-κB and JAK2/STAT3 pathways (Kang et al., 2023). Besides, Stattic ameliorates the cecal ligation and puncture-induced cardiac injury in septic mice via the IL-6-STAT3 signaling pathway (Imbaby and Hattori, 2023). These results suggest that the IL-6-STAT3 signaling pathway might be a major therapeutic target in sepsis-induced cardiac dysfunction.

### Clinical significance of our work

Sepsis remains the leading cause of admission to intensive care for cancer patients (Cooksley and Haji-Michael, 2020), and half or more of patients with malignant tumors were admitted to intensive care units due to complications with sepsis. Sepsis is more common in cancer patients than in non-cancer patients, with higher morbidity and mortality (Li et al., 2020; Liu et al., 2020b). At present, ACT001 is mainly used in the clinical treatment of cancer. Our study indicates that ACT001 may improve the immunity of cancer patients, reduce a series of side effects and sequelae caused by chemotherapy treatment, and reduce the risk of sepsis in cancer patients.

### Limitations of this study

Administrating ACT001 after CLP did not significantly prolong the survival of CLP mice in our septic shock model (all CLP mice died in 60 h after CLP). Therefore, we chose to start dosing the day before modeling. Our preliminary data showed that ACT001 can improve survival in a moderate sepsis model (20%–30% survival 5 days after CLP). This is similar to the effect of Xuebijing injection which did not significantly prolong the survival of the septic shock model when administered after CLP.

As the first step to characterize the influence of ACT001 on sepsis, we only focused on the effects of ACT001 on the circulation system. The effects of ACT001 on the immune system, respiration system, and other organs remain to be revealed. ACT001 was orally administered in clinical trials. However, its water-soluble character allowed us to test its effect by tail-vein injection. Since ACT001 can be administered orally and intravenously, this may add value to the management of sepsis and septic shock. Patients who cannot take the medicine orally may benefit from an intravenous injection of ACT001. We plan to compare the effect of orally administered and tail-vein-injected ACT001 on sepsis in the future.

## Conclusion and future direction

Our study revealed that ACT001 rescued mice from septic shock and protected cardiovascular function during sepsis. ACT001 may alleviate sepsis-induced cardiac dysfunction partially by down-regulating IL6-STAT3 signaling. Overall, these results indicated that ACT001 has a potential application in sepsis management by maintaining homeostasis of the circulation system. Some questions remain to be answered, such as whether MCL shows a similar effect on Stat3 as ACT001 in sepsis management. After oral administration, ACT001 can be rapidly distributed to various tissues and organs throughout the body and can cross the blood-brain barrier (Xi et al., 2019). We plan to uncover the influence of ACT001 on the immune system, lung, brain, and other organs in different sepsis models in the future. Comparing the effect of oral administration vs. tail-vein injection of ACT001 on sepsis is also on our research agenda.

## RNA-sequencing raw data

The raw data of RNA-Sequencing for this study have been deposited in the SRA database under accession number PRJNA1012654 (https://www.ncbi.nlm.nih.gov/sra/PRJNA1012654).

## Data Availability

The datasets presented in this study can be found in online repositories. The names of the repository/repositories and accession number(s) can be found below: NCBI, PRJNA1012654.

## References

[B1] Abrams S. T. Morton B. Alhamdi Y. Alsabani M. Lane S. Welters I. D. (2019). A novel assay for neutrophil extracellular trap formation independently predicts disseminated intravascular coagulation and mortality in critically Ill patients. Am. J. Respir. Crit. Care Med. 200 (7), 869–880. 10.1164/rccm.201811-2111OC 31162936 PMC6812439

[B74] Bian J. Bao L. Gao X. Wen X. Zhang Q. Huang J. (2022). Bacteria-engineered porous sponge for hemostasis and vascularization. J. Nanobiotechnology 20 (1), 47. 10.1186/s12951-022-01254-7 35062972 PMC8780714

[B2] Carol Illa A. Baumgarten S. Danielsen D. Larsen K. Elm T. Johansen P. B. (2021). Tail vein transection bleeding model in fully anesthetized hemophilia A mice. J. Vis. Exp. 175. 10.3791/62952 34661578

[B3] Chen X. Feng Y. Shen X. Pan G. Fan G. Gao X. (2018). Anti-sepsis protection of Xuebijing injection is mediated by differential regulation of pro- and anti-inflammatory Th17 and T regulatory cells in a murine model of polymicrobial sepsis. J. Ethnopharmacol. 211, 358–365. 10.1016/j.jep.2017.10.001 28987599

[B4] Cooksley T. Haji-Michael P. (2020). Oncologic sepsis on the ICU: two decades of improving outcomes. Crit. Care Med. 48 (6), 925–926. 10.1097/CCM.0000000000004323 32433081

[B5] Frangou E. Chrysanthopoulou A. Mitsios A. Kambas K. Arelaki S. Angelidou I. (2019). REDD1/autophagy pathway promotes thromboinflammation and fibrosis in human systemic lupus erythematosus (SLE) through NETs decorated with tissue factor (TF) and interleukin-17A (IL-17A). Ann. Rheum. Dis. 78 (2), 238–248. 10.1136/annrheumdis-2018-213181 30563869 PMC6352428

[B6] Garbers C. Heink S. Korn T. Rose-John S. (2018). Interleukin-6: designing specific therapeutics for a complex cytokine. Nat. Rev. Drug Discov. 17 (6), 395–412. 10.1038/nrd.2018.45 29725131

[B7] Gavelli F. Castello L. M. Avanzi G. C. (2021). Management of sepsis and septic shock in the emergency department. Intern Emerg. Med. 16 (6), 1649–1661. 10.1007/s11739-021-02735-7 33890208 PMC8354945

[B8] Giustozzi M. Ehrlinder H. Bongiovanni D. Borovac J. A. Guerreiro R. A. Gąsecka A. (2021). Coagulopathy and sepsis: pathophysiology, clinical manifestations and treatment. Blood Rev. 50, 100864. 10.1016/j.blre.2021.100864 34217531

[B9] Groenewegen W. A. Heptinstall S. (1990). A comparison of the effects of an extract of feverfew and parthenolide, a component of feverfew, on human platelet activity *in-vitro* . J. Pharm. Pharmacol. 42, 553–557. 10.1111/j.2042-7158.1990.tb07057.x 1981582

[B10] Hawez A. Al-Haidari A. Madhi R. Rahman M. Thorlacius H. (2019). MiR-155 regulates PAD4-dependent formation of neutrophil extracellular traps. Front. Immunol. 10, 2462. 10.3389/fimmu.2019.02462 31736940 PMC6838784

[B11] He S. Zhao J. Xu X. Cui X. Wang N. Han X. (2020). Uncovering the molecular mechanism of the qiang-xin 1 formula on sepsis-induced cardiac dysfunction based on systems Pharmacology. Oxid. Med. Cell. Longev. 2020, 3815185. 10.1155/2020/3815185 32908632 PMC7474398

[B12] Hollenberg S. M. Singer M. (2021). Pathophysiology of sepsis-induced cardiomyopathy. Nat. Rev. Cardiol. 18 (6), 424–434. 10.1038/s41569-020-00492-2 33473203

[B13] Hou Y. Sun B. Liu W. Yu B. Shi Q. Luo F. (2021). Targeting of glioma stem-like cells with a parthenolide derivative ACT001 through inhibition of AEBP1/PI3K/AKT signaling. Theranostics 11 (2), 555–566. 10.7150/thno.49250 33391492 PMC7738851

[B14] Howell M. D. Davis A. M. (2017). Management of sepsis and septic shock. JAMA 317 (8), 847–848. 10.1001/jama.2017.0131 28114603

[B15] Huang M. Cai S. Su J. (2019). The pathogenesis of sepsis and potential therapeutic targets. Int. J. Mol. Sci. 20 (21), 5376. 10.3390/ijms20215376 31671729 PMC6862039

[B16] Hui L. Yao Y. Wang S. Yu Y. Dong N. Li H. (2009). Inhibition of Janus kinase 2 and signal transduction and activator of transcription 3 protect against cecal ligation and puncture induced multiple organ damage and mortality. J. Trauma 66 (3), 859–865. 10.1097/TA.0b013e318164d05f 19276765

[B17] Iba T. Levi M. Levy J. H. (2020). Sepsis-induced coagulopathy and disseminated intravascular coagulation. Semin. Thromb. Hemost. 46 (1), 89–95. 10.1055/s-0039-1694995 31443111

[B18] Iba T. Levy J. H. (2020). Sepsis-induced coagulopathy and disseminated intravascular coagulation. Anesthesiology 132 (5), 1238–1245. 10.1097/ALN.0000000000003122 32044801

[B19] Iba T. Levy J. H. Warkentin T. E. Thachil J. van der Poll T. Levi M. (2019). Diagnosis and management of sepsis-induced coagulopathy and disseminated intravascular coagulation. J. Thromb. Haemost. 17 (11), 1989–1994. 10.1111/jth.14578 31410983

[B20] Imbaby S. Hattori Y. (2023). Stattic ameliorates the cecal ligation and puncture-induced cardiac injury in septic mice via IL-6-gp130-STAT3 signaling pathway. Life Sci. 330, 122008. 10.1016/j.lfs.2023.122008 37549828

[B21] Jaffar J. Glaspole I. Symons K. Westall G. (2021). Inhibition of NF-κB by ACT001 reduces fibroblast activity in idiopathic pulmonary fibrosis. Biomed. Pharmacother. 138, 111471. 10.1016/j.biopha.2021.111471 33730605

[B22] Jiang X. Wang Y. Qin Y. He W. Benlahrech A. Zhang Q. (2017). Micheliolide provides protection of mice against *Staphylococcus aureus* and MRSA infection by down-regulating inflammatory response. Sci. Rep. 7, 41964. 10.1038/srep41964 28165033 PMC5292736

[B23] Kalantary-Charvadeh A. Sanajou D. Hemmati-Dinarvand M. Marandi Y. Khojastehfard M. Hajipour H. (2019). Micheliolide protects against doxorubicin-induced cardiotoxicity in mice by regulating PI3K/akt/NF-kB signaling pathway. Cardiovasc Toxicol. 19 (4), 297–305. 10.1007/s12012-019-09511-2 30835049

[B24] Kang S. Tanaka T. Inoue H. Ono C. Hashimoto S. Kioi Y. (2020). IL-6 trans-signaling induces plasminogen activator inhibitor-1 from vascular endothelial cells in cytokine release syndrome. Proc. Natl. Acad. Sci. U. S. A. 117 (36), 22351–22356. 10.1073/pnas.2010229117 32826331 PMC7486751

[B25] Kang X. F. Lu X. L. Bi C. F. Hu X. D. Li Y. Li J. K. (2023). Xuebijing injection protects sepsis induced myocardial injury by mediating TLR4/NF-κB/IKKα and JAK2/STAT3 signaling pathways. Aging (Albany NY) 15 (16), 8501–8517. 10.18632/aging.204990 37650558 PMC10496990

[B26] Kato H. Hagihara M. Asai N. Umemura T. Hirai J. Mori N. (2023). Efficacy and safety of recombinant human soluble thrombomodulin in patients with sepsis-induced disseminated intravascular coagulation - a meta-analysis. Thromb. Res. 226, 165–172. 10.1016/j.thromres.2023.05.009 37182388

[B28] Lei W. Liu D. Sun M. Lu C. Yang W. Wang C. (2021). Targeting STAT3: a crucial modulator of sepsis. J. Cell. Physiol. 236 (11), 7814–7831. 10.1002/jcp.30394 33885157

[B29] Li Q. Sun Y. Liu B. Li J. Hao X. Ge W. (2020). ACT001 modulates the NF-κB/MnSOD/ROS axis by targeting IKKβ to inhibit glioblastoma cell growth. J. Mol. Med. Berl. 98 (2), 263–277. 10.1007/s00109-019-01839-0 31901951

[B30] Li W. Li Y. Qin K. Du B. Li T. Yuan H. (2019). Siglec-G deficiency ameliorates hyper-inflammation and immune collapse in sepsis via regulating src activation. Front. Immunol. 10, 2575. 10.3389/fimmu.2019.02575 31781099 PMC6859834

[B31] Li W. Nieman M. Sen Gupta A. (2016). Ferric chloride-induced murine thrombosis models. J. Vis. Exp. 115, 54479. 10.3791/54479 PMC5091988 27684194

[B32] Liu Q. Zhang S. Zhu D. Tang X. Che Y. Feng X. (2020a). The parthenolide derivative ACT001 synergizes with low doses of L-DOPA to improve MPTP-induced Parkinson's disease in mice. Behav. Brain Res. 379, 112337. 10.1016/j.bbr.2019.112337 31697983

[B33] Liu W. Chen X. Wang Y. Chen Y. Chen S. Gong W. (2019). Micheliolide ameliorates diabetic kidney disease by inhibiting Mtdh-mediated renal inflammation in type 2 diabetic db/db mice. Pharmacol. Res. 150, 104506. 10.1016/j.phrs.2019.104506 31669149

[B34] Liu Y. Wang L. Liu J. Xie X. Hu H. Luo F. (2020b). Anticancer effects of ACT001 via NF-κB suppression in murine triple-negative breast cancer cell line 4T1. Cancer Manag. Res. 12, 5131–5139. 10.2147/CMAR.S244748 32617021 PMC7326172

[B36] Lyu M. Cui Y. Zhao T. Ning Z. Ren J. Jin X. (2018b). Tnfrsf12a-Mediated atherosclerosis signaling and inflammatory response as a common protection mechanism of shuxuening injection against both myocardial and cerebral ischemia-reperfusion injuries. Front. Pharmacol. 9, 312. 10.3389/fphar.2018.00312 29681850 PMC5897438

[B37] Lyu M. Zhou Z. Wang X. Lv H. Wang M. Pan G. (2018a). Network pharmacology-guided development of a novel integrative regimen to prevent acute graft-vs.-host disease. Front. Pharmacol. 9, 1440. 10.3389/fphar.2018.01440 30618740 PMC6300759

[B38] Malaver E. Romaniuk M. A. D'Atri L. P. Pozner R. G. Negrotto S. Benzadon R. (2009). NF-kappaB inhibitors impair platelet activation responses. J. Thromb. Haemost. 7, 1333–1343. 10.1111/j.1538-7836.2009.03492.x 19566544

[B39] Mao J. Y. Zhang J. H. Cheng W. Chen J. W. Cui N. (2021). Effects of neutrophil extracellular traps in patients with septic coagulopathy and their interaction with autophagy. Front. Immunol. 12, 757041. 10.3389/fimmu.2021.757041 34707618 PMC8542927

[B40] Martin L. Derwall M. Al Zoubi S. Zechendorf E. Reuter D. A. Thiemermann C. (2019). The septic heart: current understanding of molecular mechanisms and clinical implications. Chest 155 (2), 427–437. 10.1016/j.chest.2018.08.1037 30171861

[B41] Massion P. B. Peters P. Ledoux D. Zimermann V. Canivet J. L. Massion P. P. (2012). Persistent hypocoagulability in patients with septic shock predicts greater hospital mortality: impact of impaired thrombin generation. Intensive Care Med. 38 (8), 1326–1335. 10.1007/s00134-012-2620-2 22735856

[B42] McDonald B. Davis R. P. Kim S. J. Tse M. Esmon C. T. Kolaczkowska E. (2017). Platelets and neutrophil extracellular traps collaborate to promote intravascular coagulation during sepsis in mice. Blood 129 (10), 1357–1367. 10.1182/blood-2016-09-741298 28073784 PMC5345735

[B43] Napolitano L. M. (2018). Sepsis 2018: definitions and guideline changes. Surg. Infect. (Larchmt) 19 (2), 117–125. 10.1089/sur.2017.278 29447109

[B44] Østergaard H. Lund J. Greisen P. J. Kjellev S. Henriksen A. Lorenzen N. (2021). A factor VIIIa-mimetic bispecific antibody, Mim8, ameliorates bleeding upon severe vascular challenge in hemophilia A mice. Blood 138 (14), 1258–1268. 10.1182/blood.2020010331 34077951 PMC8499050

[B45] Patel P. Walborn A. Rondina M. Fareed J. Hoppensteadt D. (2019). Markers of inflammation and infection in sepsis and disseminated intravascular coagulation. Clin. Appl. Thromb. Hemost. 25, 1076029619843338. 10.1177/1076029619843338 30991817 PMC6714897

[B46] Qin X. Jiang X. Jiang X. Wang Y. Miao Z. He W. (2016). Micheliolide inhibits LPS-induced inflammatory response and protects mice from LPS challenge. Sci. Rep. 6, 23240. 10.1038/srep23240 26984741 PMC4794649

[B47] Rittirsch D. Huber-Lang M. S. Flierl M. A. Ward P. A. (2009). Immunodesign of experimental sepsis by cecal ligation and puncture. Nat. Protoc. 4 (1), 31–36. 10.1038/nprot.2008.214 19131954 PMC2754226

[B73] Shang T. Guo Y. Li X. R. Zhou Z. Qi Y. Salahdiin K. (2022). The combination of four main components in Xuebijing injection improved the preventive effects of Cyclosporin A in acute graft-versus-host disease mice by protecting intestinal microenvironment Biomed. Pharmacother. 148, 112675. 35183993 10.1016/j.biopha.2022.112675

[B49] Sillen M. Declerck P. J. (2021). A narrative review on plasminogen activator inhibitor-1 and its (patho) physiological role: to target or not to target? Int. J. Mol. Sci. 22 (5), 2721. 10.3390/ijms22052721 33800359 PMC7962805

[B50] Singer M. Deutschman C. S. Seymour C. W. Shankar-Hari M. Annane D. Bauer M. (2016). The third international consensus definitions for sepsis and septic shock (Sepsis-3). JAMA 315 (8), 801–810. 10.1001/jama.2016.0287 26903338 PMC4968574

[B51] Sollberger G. Tilley D. O. Zychlinsky A. (2018). Neutrophil extracellular traps: the biology of chromatin externalization. Dev. Cell. 44 (5), 542–553. 10.1016/j.devcel.2018.01.019 29533770

[B52] Stoikou M. Grimolizzi F. Giaglis S. Schäfer G. van Breda S. V. Hoesli I. M. (2017). Gestational diabetes mellitus is associated with altered neutrophil activity. Front. Immunol. 8, 702. 10.3389/fimmu.2017.00702 28659928 PMC5469883

[B53] Strich J. R. Heil E. L. Masur H. (2020). Considerations for empiric antimicrobial therapy in sepsis and septic shock in an era of antimicrobial resistance. J. Infect. Dis. 222 (2), S119–S131. 10.1093/infdis/jiaa221 32691833 PMC7372215

[B54] Tiru B. DiNino E. K. Orenstein A. Mailloux P. T. Pesaturo A. Gupta A. (2015). The economic and humanistic burden of severe sepsis. Pharmacoeconomics 33 (9), 925–937. 10.1007/s40273-015-0282-y 25935211

[B55] Tong L. Li J. Li Q. Wang X. Medikonda R. Zhao T. (2020). ACT001 reduces the expression of PD-L1 by inhibiting the phosphorylation of STAT3 in glioblastoma. Theranostics 10 (13), 5943–5956. 10.7150/thno.41498 32483429 PMC7254983

[B56] Vincent J. L. De Backer D. (2013). Circulatory shock. N. Engl. J. Med. 369 (18), 1726–1734. 10.1056/NEJMra1208943 24171518

[B57] Wang C. Liu N. Yang H. T. (2020). Desflurane pretreatment can reduce sepsis-evoked lung injury in rats via inhibiting STAT3 pathway. J. Biol. Regul. Homeost. Agents 34 (3), 935–942. 10.23812/20-173-A-48 32693566

[B58] Wang M. Li Q. (2015). Parthenolide could become a promising and stable drug with anti-inflammatory effects. Nat. Prod. Res. 29 (12), 1092–1101. 10.1080/14786419.2014.981541 25429885

[B59] Wang X. T. Peng Z. An Y. Y. Shang T. Xiao G. He S. (2021). Paeoniflorin and hydroxysafflor yellow A in xuebijing injection attenuate sepsis-induced cardiac dysfunction and inhibit proinflammatory cytokine production. Front. Pharmacol. 11, 614024. 10.3389/fphar.2020.614024 33986658 PMC8112230

[B60] Wen J. J. Williams T. P. Cummins C. B. Colvill K. M. Radhakrishnan G. L. Radhakrishnan R. S. (2020). Effect of mitochondrial antioxidant (Mito-TEMPO) on burn-induced cardiac dysfunction. J. Am. Coll. Surg. 232 (4), 642–655. 10.1016/j.jamcollsurg.2020.11.031 PMC8753741 33421567

[B61] Wu D. M. Li J. Shen R. Li J. Yu Y. Li L. (2022). Autophagy induced by micheliolide alleviates acute irradiation-induced intestinal injury via inhibition of the NLRP3 inflammasome. Front. Pharmacol. 12, 773150. 10.3389/fphar.2021.773150 35115927 PMC8804324

[B75] Wu S. Huang Z. Yue J. Liu D. Wang T. Ezanno P. (2015). The efficient hemostatic effect of Antarctic krill chitosan is related to its hydration property. Carbohydr Polym. 132, 295–303. 10.1016/j.carbpol.2015.06.030 26256352

[B62] Xi X. Liu N. Wang Q. Chu Y. Yin Z. Ding Y. (2019a). ACT001, a novel PAI-1 inhibitor, exerts synergistic effects in combination with cisplatin by inhibiting PI3K/AKT pathway in glioma. Cell. Death Dis. 10 (10), 757. 10.1038/s41419-019-1986-2 31591377 PMC6779874

[B63] Xi X. N. Liu N. Wang Q. Q. Wu H. T. He H. B. Wang L. L. (2019b). Pharmacokinetics, tissue distribution and excretion of ACT001 in Sprague-Dawley rats and metabolism of ACT001. J. Chromatogr. B Anal. Technol. Biomed. Life Sci. 1104, 29–39. 10.1016/j.jchromb.2018.11.004 30423524

[B64] Xiao G. Lyu M. Wang Y. He S. Liu X. Ni J. (2019). Ginkgo flavonol glycosides or ginkgolides tend to differentially protect myocardial or cerebral ischemia-reperfusion injury via regulation of TWEAK-fn14 signaling in heart and brain. Front. Pharmacol. 10, 735. 10.3389/fphar.2019.00735 31333457 PMC6624656

[B65] Xu S. Pan X. Mao L. Pan H. Xu W. Hu Y. (2020). Phospho-Tyr705 of STAT3 is a therapeutic target for sepsis through regulating inflammation and coagulation. Cell. Commun. Signal 18 (1), 104. 10.1186/s12964-020-00603-z 32641132 PMC7341624

[B66] Yarmohammadi F. Hayes A. W. Karimi G. (2021). Natural compounds against cytotoxic drug-induced cardiotoxicity: a review on the involvement of PI3K/Akt signaling pathway. J. Biochem. Mol. Toxicol. 35 (3), e22683. 10.1002/jbt.22683 33325091

[B67] Zhai J. Guo Y. (2016). Paeoniflorin attenuates cardiac dysfunction in endotoxemic mice via the inhibition of nuclear factor-κB. Biomed. Pharmacother. 80, 200–206. 10.1016/j.biopha.2016.03.032 27133057

[B68] Zhang Q. Lu Y. Ding Y. Zhai J. Ji Q. Ma W. (2012). Guaianolide sesquiterpene lactones, a source to discover agents that selectively inhibit acute myelogenous leukemia stem and progenitor cells. J. Med. Chem. 55 (20), 8757–8769. 10.1021/jm301064b 22985027

[B69] Zhang T. Lin C. Wu S. Jin S. Li X. Peng Y. (2022). ACT001 inhibits TLR4 signaling by targeting Co-receptor MD2 and attenuates neuropathic pain. Front. Immunol. 13, 873054. 10.3389/fimmu.2022.873054 35757727 PMC9218074

[B71] Zhong J. Gong W. Chen J. Qing Y. Wu S. Li H. (2018). Micheliolide alleviates hepatic steatosis in db/db mice by inhibiting inflammation and promoting autophagy via PPAR-γ-mediated NF-кB and AMPK/mTOR signaling. Int. Immunopharmacol. 59, 197–208. 10.1016/j.intimp.2018.03.036 29656210

[B72] Zhu J. Tang C. Cong Z. Yuan F. Cai X. Yang J. (2021). ACT001 reverses resistance of prolactinomas via AMPK-mediated EGR1 and mTOR pathways. Endocr. Relat. Cancer 29 (2), 33–46. 10.1530/ERC-21-0215 34821219

