# Supplementary material for: ACT001 improved cardiovascular function in septic mice by inhibiting the production of proinflammatory cytokines and the expression of JAK-STAT signaling pathway
Source: Front Pharmacol. 2023 Nov 29;14:1265177. doi: 10.3389/fphar.2023.1265177 (PMC10716238; doi:10.3389/fphar.2023.1265177)
Supplement: Supplementary file 1 [file Table1.DOCX]

https://we.tl/t-lSp9eFq4UI
